# Suicide prevention and mood disorders: Self‐exclusion agreements for firearms as a suicide prevention strategy

**DOI:** 10.1111/appy.12455

**Published:** 2021-03-08

**Authors:** Melvin G. McInnis, Stephen B. Thompson, Sofia D. Merajver, Carl E. Schneider

**Affiliations:** ^1^ Department of Psychiatry University of Michigan School of Medicine Ann Arbor Michigan USA; ^2^ Departments of Internal Medicine and Epidemiology University of Michigan Medical School and School of Public Health Ann Arbor Michigan USA; ^3^ University of Michigan Law School Ann Arbor Michigan USA

**Keywords:** depression, guns, self‐exclusion, suicide

## Abstract

Suicide involves a complex set of behaviors and emotions that lead up to actions that may be based on planning and forethought or the result of impulse. While there are a host of antecedent circumstances the presence of a mood disorder, primarily depression, is the most common factor in suicide. While management of depression is recognized as important prevention strategy in depression, the means by which suicide occurs must be a critical element of prevention. Policies that lower access to the means for suicide will decrease the fatality. Guns are associated with half of suicides and the case fatality rate of gun associated suicide is over 90% compared to 7% for all other means. This emphasizes the importance of offering strategies that limit access to guns to those at higher risk for suicide. A declaration of formal self‐exclusion for access to firearms (guns and ammunition) offers the individual at greater risk for suicide to place themselves on an official list that would prevent them from purchasing lethal weapons. A person with depression, when well, might wish to enroll voluntarily to prevent themselves, when ill, from procuring a weapon to harm themselves or others. This recognizes the autonomy of the person and protects both the individual, the family, and society.

Suicide, willfully ending one's own life, represents one of the greatest human tragedies. There are few words in any language that describe the complex and devastating emotional aftermath of a suicide. Suicide is a worldwide problem that is often shrouded by cultural influences that inhibit the accurate record in some societies. Frequently, suicides are recorded as death by accidental injury, many involve guns. Individuals with mental health disorders are at far greater risk for suicide; depression being the most common symptoms in the background of a person who died by suicide (Kamali et al., 2019). Those with mental health disabilities die at a rate far greater that the general population (Black et al., 1985), and suicide accounts for a substantial portion of the nonnatural causes of death (Ruschena et al., 1998). Guns account for half of suicide deaths (Hemenway, 2019). Worldwide suicide trends reveal sensitivities to crisis conditions; suicides increased in many societies following the global financial crisis of 2008 (Alicandro et al., 2019). This raises concerns for the current COVID‐19 pandemic and the inherent risks to suicide that this crisis confer (Gunnell et al., 2020).

The capacity of medical professionals to predict suicidal risk is low, and the accuracy of predicting a future suicide event is essentially zero (Belsher et al., 2019). Strategies are needed to address the needs of those in crisis, whether it be the current COVID‐19 crisis or future and as yet unknown crises. Those with mental health illness continue to be at the highest risk for suicide; their circumstances are compounded by the consequences of their illness and its complications of unstable social conditions, financial stressors, co‐morbid substance abuse, and stigma‐related social perceptions. Impulse, driven by ready access to means to kill oneself, can be deadly. Gun‐related suicidal behavior has a case fatality rate (CFR) of 90% compared to a CFR of 7% for nonfirearm suicide behavior (Hemenway, 2019), providing a compelling argument for a focused effort on prevention and policy related to guns in society.

The current social upheaval from the combined stressors of the COVID‐19 pandemic and the protests over the pervasive racial injustices unmasked by multiple murders of black people has compounded an American epidemic of gun violence. There remains a divided and diverse public reaction to the public health crisis of firearms and violence. Overall, it raises the national angst, and, historically, this has led to an increase in gun‐seeking and related behaviors (Callcut et al., 2019). These viral‐like phenomena are perpetuated by the false belief that owning a gun makes one safer (Moyer, 2017). In fact, owning a gun raises the risk of personal injury, that suicide attempts are successful (Anestis, 2018), or that children's curiosity proves fatal (Anglemyer et al., 2014). Furthermore, murders of women are significantly associated with the availability of guns (Smucker et al., 2018). These facts are largely ignored, and public support for changes to firearm laws waxes then wanes with each mass shooting or critical event. Life goes on. A recent review of public opinion finds strong support for regulations banning people likely to use a gun violently from obtaining one (Haner et al., 2019). Among those considered more likely to use guns violently are the mental ill, and multivariate analytical modeling shows that gun ownership far exceeds mental illness as a predictor of firearm‐related death (Bangalore & Messerli, 2013). It is however blatantly clear that the volatile combination of guns and mental illness can be deadly (McGinty, 2018; Swanson et al., 2015). How can we empower safe self‐care strategies among those with mental illness when seeking firearms goes “viral?” Is there a roll for self‐exclusion from firearms?

Access to guns in an impulsive moment is a major factor in suicides (Anestis, 2018) which alone account for over half of firearm deaths (Swanson et al., 2015). Health care providers (and prudent friends and families) urge individuals with psychiatric illness and elevated risk of suicide to remove their access to guns. Families are advised to be proactive in denying access to guns, e.g. putting them in a secure location. Such advice is responsible clinical care and a fundamental public health obligation to the community (McGinty et al., 2014). It has been challenged with legislation attempting to interfere with the patient–doctor relationship, but such “gag laws” have been condemned by leading medical and legal societies in the U.S. (Jones et al., 2018; Weinberger et al., 2015). Indeed, the conversation is better centered around developing effective and standardized ways of talking with patients about safety and guns (Betz & Wintemute, 2015; Damari et al., 2018). It is simply a basic human right to be cared for when ill, and a health care provider's duty to advise patients to minimize dangers in their personal environment.

Predicting violence is challenging and all but abandoned by the criminal justice system (Monahan, 2018) as consequences are based on fact not projected risks; identifying those likely to use guns violently is also notoriously difficult (Fazel et al., 2012). Complicating the laws surrounding guns and gun use is the need to avoid infringing constitutional rights and to avoid exacerbating the stigma of mental illnesses (thus discouraging those who need care from seeking it) (Appelbaum & Swanson, 2010). Depression, bipolar disorder, and other medical and psychiatric diseases often impair judgment and *predispose* sufferers to act impulsively (Swann et al., 2005), with the *potential* for lethally harming themselves and others (Anestis, 2018). The overwhelming majority of people with these diseases will never use a gun violently (Steadman et al., 2015; Swanson et al., 1990); however, suicidal thoughts are often part of significant depression, and depression is a common and powerful risk factor for suicide (Anglemyer et al., 2014). The risks are real.

In the current charged, high stakes public‐health crisis (Webster & Wintemute, 2015), an opportunity exists to avoid the potentially disastrous results of gun ownership by vulnerable individuals. While many legislated directives formally and effectively restrict gun ownership based on adjudicated criteria (Zeoli & Webster, 2019), the opportunity for others at risk for harmful use of guns to make a personal commitment against gun ownership should be offered. Such an opt‐out is not new gun control legislation. It is simply aligned with current perspectives of self‐determination in personal health and individual autonomy.

Since 1998, the U.S. Department of Justice, through its Federal Bureau of Investigation, has maintained the National Instant Criminal Background Check System (NICS), which can run a background check on any person seeking to purchase a firearm from a licensed retailer (Justice, 2014). When the required information identifying the prospective purchaser is submitted, the NICS checks databases of federal and state information that would prevent a person from legally buying a gun. Within minutes, the NICS returns one of three answers: “proceed,” “deny,” or “delay” for further investigation. The NICS decision to deny an attempted purchase does not identify the reason for the denial and does not imply a criminal record. It is, however, a robust and reliable method of excluding individuals from purchasing firearms through a licensed retailer.

A sensible use of the NICS database is to register the personal request to receive the “deny” response should the individual try to purchase a firearm (Vars, 2015). This personal choice and commitment, resembling a “Ulysses contract” (Ulysses tied himself to the mast so that the Sirens' alluring song would not seduce him into driving his ship onto the rocks), is not new. Some states allow people to put themselves on a list excluding them from gambling establishments (NJ Office of the Attorney General, 2015). The medication disulfiram can be prescribed to, and voluntarily taken by, those who want help resisting the impulse to drink alcohol (Duckert & Johnsen, 1987). Living wills allow individuals to make advance choices that are to be enforced when the person loses the competence to make medical decisions. A voluntary self‐exclusion for firearm purchase is a logical extension of modern society's respect for autonomy and will help those predisposed to impulsive decisions to protect themselves, their loved ones, and their neighbors.

Voluntary exclusions from access to firearms honor personal choices yet raise several questions. Should society allow people who have a history of suicide behavior to purchase and own guns? Would an exclusion policy impede individuals from seeking help for psychiatric illness? Will the Department of Justice honor a self‐exclusion request? Should someone with documented mood or emotional instability be encouraged to consider self‐exclusion? Should a period of self‐exclusion be indefinite or last a defined period? Should self‐exclusion be subject to later revocation, and if so, what procedures would govern that revocation? Does revocable self‐exclusion simply amount to a de facto waiting period to purchase a firearm (Vars, 2015)?

The common good that voluntary firearm self‐exclusion may do in diminishing death and distress is substantial. Several states in the USA provide for a short‐term, court‐ordered involuntary restriction on gun purchase and possession, and studies from Connecticut (Swanson et al., 2017) and Indiana (Swanson et al., 2019) suggest that for every 10 gun‐removal actions, one life was saved. Entrusting friends with one's firearms is certainly an easy option, and care providers should identify opportunities when they are concerned for health and safety (Pallin et al., 2019). Empowering individuals to formalize such a request in the court systems is very attractive as it provides a formal agreement with official powers to prevent acquiring firearms. The cost is vanishingly low and could be implemented on a national basis. It provides the opportunity for the vulnerable individual to exercise their health‐centered and personal right to self‐determination by denying themselves access to firearms.

## CONFLICT OF INTEREST

The authors declare no conflict of interest related to this manuscript.

## Data Availability

Data sharing is not applicable to this article as no new data were created or analyzed in this study.
